# Atención Domiciliaria y Pandemia Covid-19: Experiencia Desde Enfermeria

**DOI:** 10.15649/cuidarte.1980

**Published:** 2021-08-20

**Authors:** Jeimmy Johana Blanco Caviedes, Angela Maria Henao-Castaño, Martha Esperanza Ovalle Garzón

**Affiliations:** 1 Grupo Cuidar SAS, Colombia. Email: jeimmyblancocaviedes@ gmail.com Grupo Cuidar SAS Colombia jeimmyblancocaviedes@ gmail.com; 2 Universidad Nacional de Colombia Bogota, Colombia. E-mail: angmhenaocas@unal.edu.co Autor de correspondência Universidad Nacional de Colombia Universidad Nacional de Colombia Colombia; 3 Grupo Cuidar SAS, Colombia. Email: martaespe6@hotmail.com Grupo Cuidar SAS Colombia martaespe6@hotmail.com

**Keywords:** Enfermería, Infecciones por Coronavirus, Visita Domiciliaria, Investigación Cualitativa., Nursing, Coronavirus Infections, Home Visit, Qualitative Research., Enfermagem, Infecções por Coronavírus, Visita Domiciliar, Pesquisa Qualitativa.

## Abstract

**Introducción:**

La atención domiciliaria busca brindar una solución a los problemas de salud en el domicilio con el apoyo de profesionales, técnicos del área de la salud y la participación de la familia, en tiempos de pandemia por COVID-19 se deben ajustar las dinámicas a este nuevo contexto

**Objetivo:**

Comprender el significado de la experiencia de enfermería brindando cuidado en atención domiciliaria en época de pandemia

**Materiales y Métodos:**

Estudio fenomenológico hermenéutico que incluyó a 15 enfermeros que laboran en una institución de hospitalización domiciliaria. Se realizaron entrevistas en profundidad, se analizó la información y se generaron las categorías que representan la experiencia

**Resultados:**

El análisis fenomenológico arrojó las siguientes categorías existenciales. El distanciamiento social un cambio en la cotidianidad, La prevención del contagio es responsabilidad de todos y desafíos para el paciente y familia en el domicilio.

**Conclusión:**

El personal de enfermería debe ser responsable del cumplimento de los protocolos de bioseguridad y por el bien de ellos mismos, sus pacientes y familiares, el trabajo en equipo y la educación permanente con la escucha activa hacen que esta pandemia por la que atraviesa el mundo no deteriore el cuidado de los pacientes que requieren cuidado en los domicilios.

## Abstract (en)

**Introduction:**

Home-based care seeks to provide a solution to home health care issues supported by healthcare professionals, practitioners and the participation of the family, whose dynamics in COVID-19 times should be adjusted to this new context.

**Objective:**

To understand the significance of the nursing experience providing care in home-based care settings in pandemic times.

**Materials and Methods:**

A hermeneutic-phenomenological study was conducted with 15 nurses who work in a home care service provider. In-depth interviews were conducted from which the information was analyzed and classified into categories representing the experiences.

**Results:**

The following categories were obtained from the phenomenological analysis: social distancing as a change in daily life, preventing COVID-19 infection is everyone’s responsibility and home care challenges for patients and families.

**Conclusions:**

Nursing professionals must ensure compliance with biosafety protocols and for the good of themselves, their patients and families, teamwork and continuing education with active listening ensure that home care of patients does not deteriorate in these pandemic times

## Resumo

**Introdução:** A atenção domiciliar busca dar uma solução aos problemas de saúde no domicílio com o apoio de profissionais, técnicos da área da saúde e a participação da família, em tempos de pandemia pela COVID-19, a dinâmica deve ser ajustada a este novo contexto.

**Objetivo:**

Compreender o significado da experiência da enfermagem no cuidado na atenção domiciliar em tempos de pandemia.

**Materiais e Métodos:**

Estudo fenomenológico hermenêutico que incluiu 15 enfermeiras que trabalham em uma instituição de internação domiciliar. Foram realizadas entrevistas em profundidade, analisadas as informações e foram geradas as categorias que representam a experiência.

**Resultados:**

A análise fenomenológica gerou as seguintes categorias existenciais. O distanciamento social uma mudança no cotidiano, a prevenção do contágio é responsabilidade de todos e desafios para o paciente e família em casa.

**Conclusão:**

A equipe de enfermagem deve se responsabilizar pelo cumprimento dos protocolos de biossegurança e para o bem de si próprios, de seus pacientes e familiares, o trabalho em equipe e a educação permanente com escuta ativa fazem com que essa pandemia na qual o mundo atravessa não prejudique o atendimento aos pacientes que necessitam de cuidados domiciliares.

## Introducción

La atención domiciliaria constituye un conjunto de actividades de carácter socio-sanitario y de ámbito comunitario, que se realiza en el domicilio de la persona con la finalidad de detectar, valorar, dar apoyo y hacer un seguimiento de la persona con problemas de salud y de su familia potenciando su autonomía y la calidad de vida(1). El Ministerio de Protección Social lo define como una modalidad de prestación de servicios de salud extra hospitalaria que busca brindar una solución a los problemas de salud en el domicilio o residencia y que cuenta con el apoyo de profesionales, técnicos o auxiliares del área de la salud y la participación de la familia(2). En los últimos años se ha despertado nuevamente el interés por la atención en domicilio, forzados por motivos económicos, y por el aumento de las enfermedades crónicas(3). El reto de la atención a domicilio consiste en que sea capaz de proporcionar al paciente una asistencia sanitaria con calidad y calidez(4).

Algunos antecedentes de la atención domiciliaria la presentan como un programa que se restringía básicamente a personas que no contaban con un familiar que le brindara cuidados, o favores religiosos; cuyas características se enfocaban en las necesidades que tenía el paciente y si estas podían cubrirse en el domicilio, además de realizar un análisis de costo beneficio particularizando cada caso(5).

Desde el soporte empírico se describen diferentes demarcaciones que se deben tener en cuenta cuando se habla de la atención domiciliaria resaltando que esta atención debe ser continua, longitudinal además debe contar con el apoyo de un grupo interdisciplinario y resaltando la inclusión de la familia en plan de cuidados que se deben efectuar en el momento de iniciar un servicio de atención domiciliaria(6).

Por ello la atención en el domicilio tiene como particularidad principal el abordaje de la consulta de enfermería fuera del entorno sanitario, haciendo uso de métodos directos de valoración tales como: Valoración de la capacidad funcional física, de las actividades de la vida diaria, valoración cognitiva y funcionamiento familiar entre otros. La asistencia sanitaria en el domicilio del paciente tiene múltiples ventajas ante la posibilidad de una internación en un hospital o institucionalización, mantener al paciente en su medio más cercano y habitual su entorno familiar. Facilitar la realización de actividades orientadas a favorecer la inserción y rehabilitación del paciente en su medio habitual y familiar.

Es importante conocer la función que cumple el personal de enfermería en el domicilio, cuyo objetivo es mejorar la calidad de vida del paciente y cumplir con un plan de cuidadosqueseestablecensegúnlanecesidaddelmismo, entre las que encontramos el apoyo no solamente de las necesidades básicas como asistencia en alimentación, aseo y administración de medicamentos si no que se vuelven una parte fundamental en el apoyo a la rehabilitación y estabilización del paciente tanto física como emocional.

Por lo tanto, en estos momentos de la pandemia COVID-19 se han generado cambios en todas las esferas de las personas y el grupo de enfermería no es la excepción(7). El personal de enfermería que labora en las instituciones prestadores de salud domiciliaria se enfrenta a una situación que precisa del confinamiento domiciliario de personas sanas, pero susceptibles de contagio, conviviendo con personas contagiadas y sus cuidadoras en situación de aislamiento. Se recomienda como estrategia de adaptación a la pandemia que la atención domiciliaria reduzca las visitas presenciales en el domicilio y priorizarse por el riesgo de contagio que conlleva para el grupo de enfermería, reduciéndolas a aquellos casos imprescindibles en los que haya que realizar valoración, exploración y actuación(8).

De cualquier forma, el personal que se integra al cuidado de pacientes con alto riesgo de contagio o contagiados se enfrenta a dificultades bioéticas como lo son el cuidar un paciente positivo afrontando el riesgo de poder contagiarse y poner en riesgo a su propia familia frente al hecho de cumplir con la misión que le ha sido asignada.

Por otra parte, el adulto mayor con enfermedad crónica es la población que mayor demanda de este servicio y durante la pandemia las personas mayores son consideradas con una alta vulnerabilidad por la letalidad de la enfermedad. Unido a esto durante la pandemia, los servicios de salud se han visto obligados a reorganizarse priorizando la atención de pacientes graves con COVID-19 y dejando en un segundo plano el cuidado de pacientes con otras enfermedades. Esta reorganización se ha puesto de manifiesto principalmente en forma de retrasos en el diagnóstico de las enfermedades, así como demoras, modificaciones o interrupciones en el tratamiento farmacológico, quirúrgico o de otro tipo, haciendo que la atención domiciliaria sea el único servicio de salud de estos pacientes(9).

Sin embargo, se encuentran pacientes que requieren de atención continua en la cual no es una opción disminuir los cuidados de enfermería exponiendo al riesgo de contagio a los enfermeros, los pacientes y sus familias, fue esta situación la que llevo a plantear el siguiente estudio con el objetivo de comprender el significado de la experiencia del grupo de enfermería durante el cuidado en atención domiciliaria en tiempos de pandemia.

Es por ello que consideramos conveniente realizar un estudio sobre la experiencia del personal de enfermería en atención domiciliaria en tiempos de pandemia que nos ayude a entender las dificultades que deben afrontar en el plano laboral como personal.

## Materiales y Métodos

Con el fin de comprender la experiencia de enfermería brindando cuidado en atención domiciliaria en época de pandemia se realizó esta investigación cualitativa, con diseño fenomenológico. La investigación cualitativa permite desarrollar conceptos que nos ayudan a comprender los fenómenos sociales, con énfasis en significados, experiencias y puntos de vista de los participantes(10). El abordaje fenomenológico se dio bajo el referente de Martin Heidegger, cómo método para dirigir investigaciones y poder comprender en la medida de las vivencias del ser humano(11),(12). hasta llegar a la esencia de la experiencia del participante y de las relaciones entre las experiencias(13).

El estudio se realizó en una Institución prestadora de salud domiciliaria de la ciudad de Ibagué con quince años de fundada. Los participantes fueron 15 integrantes del grupo de enfermería por medio de un muestreo intencionado que cumplieran con los criterios de inclusión, como tener experiencia laboral mayor de un año en la institución y que estuvieran brindando cuidado en el domicilio durante la pandemia. Los encuentros se dieron entre los meses de mayo y junio del 2020 en las instalaciones de la institución prestadora de salud, en la sala de juntas de manera individual, los participantes fueron convocados por correo electrónico y posterior llamado telefónico con el fin de conocer su participación y agendar el horario de la entrevista, en todos los casos los participantes demostraron buena disposición para el desarrollo de esta.

Para la recolección de la información se llevó a cabo una entrevista en profundidad, cada entrevista partió de la siguiente pregunta central. ¿Cuál es el significado de la experiencia de brindar cuidado en el domicilio en época de pandemia? Fue grabada en audio, tuvo una duración de entre 25 - 30 minutos. Para valorar la capacidad de comunicación e interacción del investigador con el participante se efectuaron dos entrevistas preliminares; también se evaluó la pregunta, para identificar aquellas que generan respuestas que enriquecieron posteriormente el análisis. Las entrevistas fueron validadas por medio de la revisión de los hallazgos por expertos lo cual contribuyó a estimar en qué medida los procedimientos utilizados se ajustan a la realidad del objeto de estudio y favorecen su replicabilidad.

El análisis de la información se realizó según los pasos propuestos por Taylor.Bogda(14), 1. Preparación de los datos, que incluye la transcripción de la entrevista la cual se llevó a cabo de manera fiel, inmediatamente después de realizarla y sin omitir las frases o expresiones del participante dentro de su contexto; luego se comparó con la grabación y se guardó en medio electrónico como lo recomiendan Cohen(13). 2 Organización de los datos, la interpretación inicial arrojó los aspectos generales que resaltó el participante; siguió la codificación, el análisis y la transformación de éstos en datos significativos. 3. Interpretación, la. cual se dio con el uso del software Atlas Ti, la saturación de los datos se dio con la convergencia de los significados que emergieron.

La credibilidad de la información se llevó acabo por medio de la presentación de la información a cinco de los participantes con el fin de confirmar los hallazgos y datos particulares. La auditabilidad de los datos fue realizado por uno de los investigadores que no tuvo ningún tipo de contacto con los participantes en la recolección de la información, verificando las entrevistas y examinando los datos encontrando similitudes y algunas diferencias que se colocaron en discusión con los investigadores y posterior consenso, buscando así el rigor en la investigación(15).

El estudio incluyó todos los aspectos éticos para exponer a los participantes, el estudio conto con el aval institucional para su ejecución bajo los lineamientos de la Declaración-Helsinki(16). A cada participante se le explicaron los objetivos del estudio y la metodología escogida, para luego dar su consentimiento verbal. Cada uno pudo decidir si aceptaba participar en el estudio, en un acto voluntario y sin ofrecer remuneraciones económicas ni de otro tipo. Tampoco se ejerció ningún tipo de presión o coacción por parte del investigador, quien dio a conocer que la entrevista terminaría en el momento que ellos lo decidieran. Todas las entrevistas se llevaron a cabo sin contratiempos. Se protegió la privacidad de los participantes y la confidencialidad de su información evitando el uso de nombres propios, para lo cual se diseñó un sistema de numeración que identifica cada una de las entrevistas con la letra E.

Desde los supuestos éticos de Ezequiel Emanuel(17) en el cual el valor social y científico que otorga al estudio, está dado por el conocimiento que se aporta de un servicio extramural de gran demanda en el mundo con diferentes actores inmersos como el paciente y el cuidador formal. Así como proporción favorable del riesgo-beneficio, se contó en el equipo de investigación con la psicóloga la cual estuvo presente para realizar cualquier tipo abordaje terapéutico dada la carga alta de stress derivado de la pandemia y vulnebailidad emocional que se llegara a presentar durante la recolección de la información, no fue necesario ningún tipo de manejo psicológico en los participantes del estudio al culminar las entrevistas.

## Resultados

Para obtener una comprensión del significado de la experiencia del personal de enfermería durante el cuidado en atención domiciliaria en tiempos de pandemia se identificaron categorías aportadas por la experiencia. Inicialmente, se identificaron las características generales de los participantes, se encontró que el 94% eran mujeres y el 6% hombres con edades entre los 22 y 38 años que laboran en hospitalización domiciliaria por más de un año. Las tres categorías que emergieron del análisis de los datos fueron las siguientes.

**Figura 1:**
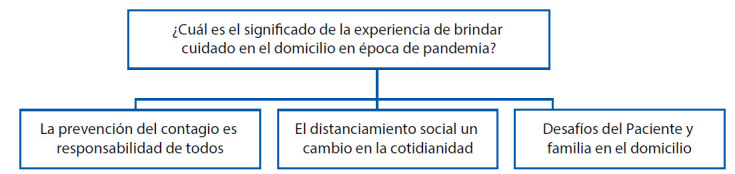
Matriz hallazgo de categorías

Fuente: Elaboración propia. Estudio 2020

### La prevención del contagio es responsabilidad de todos

En esta primera categoría los participantes reconocen que en estos momentos de pandemia COVID-19 en el mundo se enfrentan ante diferentes temores en los cuales se encuentran la salud propia, la de su familia y la de su paciente, reconocen que son un riesgo de contagio para esa persona vulnerable y que seguramente si enferma el riesgo de muerte es alto dadas las múltiples comorbilidades que presentan los pacientes en el domicilio.

*“cuidado de nosotros como también de las demás personas, se debe evitar la propagación del virus y posibles contagios, para así mantener la calidad de vida nuestra y la de otras personas también incluyendo nuestra familia” E4.*

Reconocen la importancia de dar cuidado desde las necesidades emocionales de los pacientes *“Pues en este caso las personas que viven en el domicilio y el paciente al cual se le brinda cuidado emocional durante el cuidado, pues de igual manera los pacientes, así permanezcan en el domicilio conocen lo que está pasando con la pandemia” E3*.

De una y otra manera desarrollan mecanismos adaptativos para sobrellevar la situación. *“Igual También tenemos que adaptarnos porque de eso se trata para el cuidado y bienestar de todos” E 2 “La mejor manera de adaptarnos es evitando salir cuando no estamos laborando, porque si llegamos a contagiarnos ponemos en riesgo al paciente” E14*

Teniendo en cuenta la responsabilidad de cada una de las personas en la atención domiciliaria los participantes identifican como el equipo de Bioseguridad son elementos que deben tener con un buen uso en pro del bienestar de todos.

*“Se deben tener todas las normas de bioseguridad, si me acercó a un procedimiento invasivo entonces toca usar gafas de protección tapabocas dependiendo pero que se le vaya hacer si el tapaboca convencional o tapaboca alta eficiencia, el uso de guantes y gafas” E14*

*“Esta pandemia no ha obligado a realmente tener autocuidado, no pasar por alto las normas de prevención de contagio, tener puesto todos los elementos de bioseguirdad” E7*

### El distanciamiento social un cambio en la cotidianidad

Esta segunda categoría representa como la atención domiciliaria se constituye en una opción por parte del paciente dependiente de recibir atención continua, integral y multidisciplinaria, el personal de enfermería realiza actividades de cuidado enmarcadas en dinámicas cotidianas en las cuales el distanciamiento social obligatorio se encuentra presente.

*“Todos hemos tenido el mismo impacto porque siempre se acostumbra a socializar con todas las personas que nos acompañan, el día a día nos cambió” E2.*

*“Verdad, se vive con más cuidado de seguridad con mi paciente que con mi familia en este momento”. E8*

Desde el aspecto emocional de los enfermeros los participantes expresan. *“El distanciamiento me afecta porque yo soy una persona que me gusta mucho el deporte el ejercicio y la verdad no he podido asistir al lugar que a mí me gusta” E*10.

*“la pandemia nos afectó, debido a que puedes cambiar muchas cosas que antes habitualmente estábamos acostumbrados a realizar por ejemplo el llegar a casa el saludar siempre habría un beso un abrazo para la familia para el esposo para la hija y ahorita pues ya no ya no se realiza eso primero es los otros cuidados no, lavado de manos o algo así y el distanciamiento ya no es tanto social sino también familiar” E12*

Se mantiene un gran reto en la atención domiciliaria y es relacionada con continuar cumpliendo con los lineamientos dados y seguir prestando el servicio para los pacientes y las familias dando seguridad y confianza, sin dejar de sentirse insegura y preocupada por el riesgo de exposición pero que como profesional de salud no puede manifestarlo, ni expresarlo.

Permanentemente se debe hacer un acompañamiento al personal de enfermería y educación al paciente y su familia para que ese reto sea cada vez una meta lograda, cada día proporcionando una atención humanizada y terapéutica.

### Desafíos del Paciente y familia en el domicilio

Esta tercera categoría presenta el binomio paciente y familia. No debemos olvidar que el domicilio es el hábitat natural de las personas, en él se ubica el núcleo familiar, es el sitio de referencia del paciente y llega a constituir la parte más importante de su historia personal. La familia ha enfrentado cambios en las dinámicas del domicilio propias del aislamiento social como una estrategia de prevención para adquirir la infección viral.

*“ son cuidados especiales lo mismo ahí que la familia tiene que estar atenta con todos los cuidados para evitar que lleguen visitas para evitar cualquier tipo de complicación para que le lleven el virus digámoslo así al paciente o algo o al cuidador es en la parte social de los lo que mandó el gobierno que es para la prestación de servicio porque el paciente tiene interconsultas y las interconsultas pues no se pueden hacer las citas a tiempo por decir exámenes la toma de exámenes y quemas los insumos también para reclamar los es un poquito más complejo ”E6*

La familia juega un rol de protección en cada una de las acciones del día a día, evitando las visitas en el domicilio, el aislamiento del paciente, el lavado de manos frecuente, el uso de tapabocas cuando ingresaba en la habitación. *“Muchos de los familiares se aíslan dentro de la casa evitando ingresar con frecuencia en la habitación del paciente, especialmente cuando han estado haciendo diligencias y han salido de la casa” E4*

Así mismo los pacientes manejan una rutina al interior de los domicilios lo que hace que algunas ocasiones no se vean afectados con las medidas de aislamiento social, dado que algunos ellos no realizan actividades al aire libre de manera permanente solo salen de los domicilios para los controles de consulta especializada.

*“Pues reitero que se llevan en el caso de cumplir una cita médica o de algún examen que tenga que requiera que el paciente debe prestar presente pues para realizar dicho procedimiento, mis pacientes no han presentado algún cambio emocional así impactante porque ellos ya están enseñados a esa rutina en su entorno familiar este encierro y todo lo que se ha tomado en esta pandemia” E7*

Teniendo en cuenta la situación por la que se vive en el mundo por la pandemia, los enfermeros que laboran en atención domiciliaria reconocen que se debe ser prudente con lo que se dice en el domicilio especialmente en pro de salud mental de los pacientes, creando un cambio en los mensajes que se dan a los familiares y en su cotidianidad durante la interacción.

*“Esta experiencia se debe ver de buena manera, debemos tranquilizarnos mucho o sea él no expresar digamos lo que yo siento porque al expresar lo que yo siento pondría más preocupante a la persona que está en el rededor” E9.*

*“Desde que empezó la pandemia, pienso mucho lo que les dijo a los pacientes, reciben información muy fuerte de los que está pasando y es mejor no opinar para evitar que se sientan temores” E13*

## Discusión

Estos hallazgos identifican como el domicilio se constituye en el espacio de calidez, intimidad, de acompañamiento, de comprensión permanente e incondicional, aspecto que cobra mayor importancia en el caso de pacientes con enfermedades crónicas, terminales y/o enfermedades como la actual pandemia COVID-19, enfermedad viral que genera mucha ansiedad y preocupación por ser una patología desconocida y que la ciencia hasta ahora está aprendiendo su comportamiento y manejo, cuya carga emocional y espiritual se intensifica en los últimos momentos de su vida(18)

La Organización Mundial de la Salud plantea que se deben tener medidas concertadas para mitigar su impacto en todas las dimensiones de los cuidados de larga duración, incluidas la atención domiciliaria y la atención comunitaria, dado que la mayoría de los usuarios y proveedores de cuidados son vulnerables a una COVID-19 grave. Las medidas de respuesta que se adopten en el ámbito de los cuidados de larga duración serán uno de los elementos fundamentales y decisivos para mitigar la pandemia de COVID-19 en muchos países(19). La respuesta que desde enfermería expresaron los participantes está relacionada con la responsabilidad en las medidas de bioseguridad con el uso del equipo de protección personal en pro de la salud de todos(20).

La protección para el personal de enfermería, los pacientes y sus familias, es decir mantener un trabajo en equipo y así contribuir a la seguridad del paciente, y la creación de equipos sólidos con confianza mutua y colaboración debe ser, por lo tanto, una parte esencial del trabajo de los gerentes con la seguridad del paciente. El enfoque principal a la hora de construir una buena cultura de seguridad para los pacientes debe ser la comunicación abierta, asegurando que se valoren las ideas y sugerencias del personal(21).

Esta situación ha llevado que los enfermeros de atención domiciliaria se adapten al mundo de la vida de los pacientes; la excelencia en la atención se ve amenazada por el contexto de pandemia(22). Llevando a que se reconozcan las necesidades emocionales de los pacientes y las familias y el cuidado se convierta en un vehículo de comunicación y de expresiones alentadoras con el fin de no aumentar la tensión en el ambiente.

Los estudios reportan como lo enfermeros en el domicilio buscan mejorar los procesos organizativos, la participación de los miembros de la familia y sus propias habilidades y sentido de propósito(23),(24). Presentando comportamientos de enfermería relacionados con las necesidades físicas y necesidades psicosociales del paciente.

Sin embargo, los sentimientos vividos por el personal de enfermería en el cuidado de atención domiciliaria están presentes dado el temor de contraer la infección y el aislamiento al que están sometidos llevando a cambios en las dinámicas sociales que se han tenido que tomar. Estos sentimientos de temor y stress se han reportado en China con el personal de salud que experimentó estrés emocional durante el brote de COVID-19, los factores más importantes que motivaron a seguir trabajando eran sus responsabilidades sociales, morales y obligaciones profesionales(25).

## Conclusiones

El personal de enfermería de atención domiciliaria que brinda cuidado a un paciente con patologías que conoce, realiza su trabajo de manera segura y tranquila, pero cuando el cuidado debe ser a una patología desconocida con alta posibilidad de contagio y en un paciente de gran vulnerabilidad, se ve enfrentando al paciente y familia quienes esperan continuar con el servicio pero también que no haya riesgos para ese núcleo familiar que atiende, para sí mismo y su familia, esta situación genera ansiedad y preocupación.

Dentro de las experiencias vividas la responsabilidad y trabajo en equipo del personal se caracterizaron, especialmente la que tenían con sus pacientes, el apoyo de la familia y el acompañamiento de la entidad.

Por tal motivo es de vital importancia generar políticas que favorezcan al personal de salud no solamente en lo económico si no en la protección de la salud mental y bienestar social para que puedan desarrollar sus actividades con mayor tranquilidad.
